# Rapid detection of herpes simplex virus 2: a SYBR-Green-based real-time PCR assay

**DOI:** 10.12688/f1000research.53541.1

**Published:** 2021-07-26

**Authors:** Modhusudon Shaha, Bithi Roy, Mohammad Ariful Islam

**Affiliations:** 1 Microbial Biotechnology Division, National Institute of Biotechnology, Dhaka, Bangladesh; 2 Agronomy, Bangladesh Agricultural University, Mymensingh, Mymensingh, Bangladesh; 3 Microbiology, Jagannath University, Dhaka, Bangladesh

**Keywords:** Herpes simplex virus 2, real-time PCR, rapid diagnosis, primer design

## Abstract

The prevalence of Herpes simplex virus 2 (HSV2) is increasing at an alarming rate in the world. Most of the HSV2 cases are not diagnosed properly, although a range of molecular and serological diagnoses exist. Herein, we have reported a very rapid detection method specific for HSV2 using real-time PCR. The primers specific for HSV2 were designed using the Primer-BLAST tool and 120 base pairs of the polymerase gene were amplified using real-time PCR with SYBR Green dye. The designed primer pair was found highly efficient in detecting only HSV2 DNA, but not HSV1. The threshold cycle (Ct) value for HSV2 reactions by designed primers was found to be an average of 22.55 for a standard copy number of viral DNA that may denote the efficiency of the primers. The melting temperature (Tm) of the amplicon using designed primers (82.6 ^0^C) was also higher than that using reference primers (about 78 ^0^C), indicating the high GC content of the amplified template. The designed primer pair will help clinicians to detect the HSV2 DNA specifically and diagnose the associated disease rapidly.

## Introduction

Herpes simplex virus 2 (HSV-2), a highly infectious pathogen, is a member of the Herpesviridae family consisting of a double-stranded DNA material. ^1, 2^ Globally, about 417 million people are infected by HSV-2, with a prevalence of 11.3%. ^3^ The most common age group with HSV-2 diseases is 15-49 years of age, with a slightly higher rate of infection in women. ^3^ HSV-2 infection is characterized by painful genital ulcers with vesicular rash (watery blisters), which are highly contagious and spread viruses to other hosts upon direct contact. ^4^

The diagnosis of HSV2 is complex and costly in developing countries like Bangladesh. Moreover, due to the high similarity of HSV2 with other human herpes viruses (HHV) such as HSV1, it is tough to confirm the type of the diagnosed HHV. Nowadays, molecular analysis exists to determine specific types of HHV, but identification of the virus type is solely dependent on nucleotide sequencing. In this case, developing a new real-time polymerase chain reaction (RT-PCR) method for HSV-2 diagnosis can facilitate HSV2 detection.

RT-PCR is the upgraded version of conventional PCR, invented by Kary Mullis in 1984, which aims to detect and quantify genes with dynamic expression profiles. ^5^ Analysis by RT-PCR has several important steps including DNA extraction, designing primers and amplification. ^5^ This study sheds light on the development of a RT-PCR assay to detect HSV2, which includes the design of compatible primers and amplification of HSV2 DNA using this primer set.

## Methods

### HSV2-specific primer design

Appropriate primer design is a very important step to diagnose and quantify a specific microbial gene. An important, general step to design a primer set is to ensure the optimal melting temperature (Tm), balanced GC content, self-complementarity and self-3’ complementarity. ^6^ There are several online tools to develop and design primers, such as NCBI’s Primer-BLAST. ^6^ A known gene sequence of HSV2 virus collected from NCBI GenBank (MH697422.1, see *Underlying data*) was used for Primer-BLAST in the NCBI server keeping the parameters as default.

**Figure 1.**
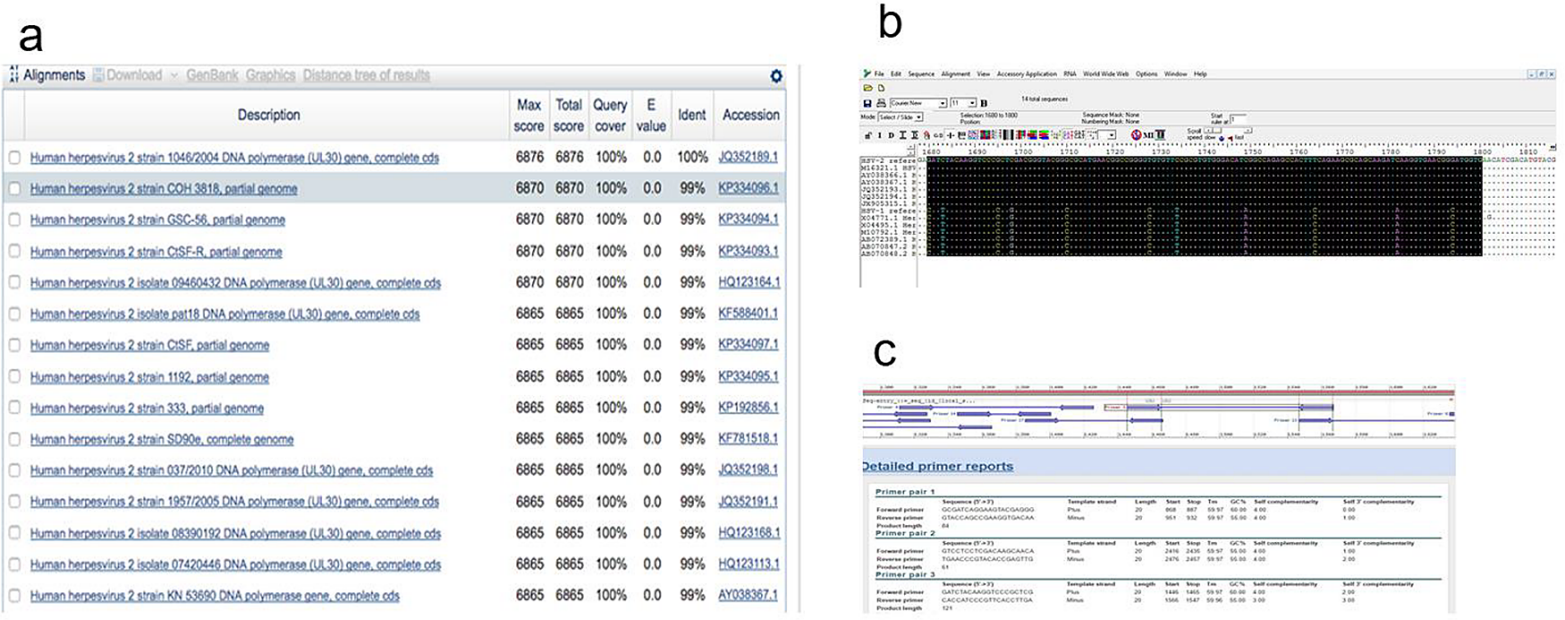
Primer design for real-time PCR specific to HSV2. (a) Collection of HSV sequences using NCBI-BLAST for designing a primer pair; (b) Alignment of HSV sequences and amplification region (selection region) using BioEdit software; (c) Different primer pair sets and associated properties; primer pair-3 was selected for further analysis.

Using the online NCBI nucleotide Basic Local Alignment Search Tool (BLAST) ^6^ several highly similar sequences of HSV1 and HSV2 were collected based on alignment score (quality of the alignment), query cover (nucleotide cover of searched sequence), Expected value (E, expected number of alignments by chance with a particular score; the lower the E value, the more significance the score) and identity with the reference sequence ( Figure 1a), and then aligned using BioEdit software version 7.2, keeping HSV2 sequences at the top ( Figure 1b). The primers obtained were then checked to find the best region to anneal with HSV2 but not HSV1 (in order to develop the primer specific for HSV2). The region with maximum nucleotide matches to the reference HSV2 sequences and minimum nucleotide matches to the HSV1 sequences was selected. Among all sets of primers tested, primer-3 was found the best regarding properties like a greater number of nucleotide mismatches with HSV1 sequences, self-complementarity (a predictor secondary structures between a single primer or primer pairs), higher Tm compared to other not-selected primers) and GC content (in good range 55-60%), and most importantly, 3’ self-complementarity (useful for predicting primer-dimers) ( Figure 1c).

### Setting up a RT-PCR reaction

The real-time PCR (based on SYBR Green fluorescent binding) with HSV1 and HSV2 template DNA were prepared using the designed primer pair (specific for HSV2) and control primer pair (common for both HSV1 and HSV2). In a 20ul reaction mix, 10ul of SYBR Green Master Mix (2X), 1ul of each reverse and forward primers at 6pM concentrations, 6ul of nuclease-free water and 2ul of template HSV2 DNA were added. The reagents were purchased from Qiagen (Germantown, MD, USA). The reaction conditions used to amplify the DNA were an initial 95°C for 20 seconds, 40 cycles of 3 seconds at 95°C and 30 seconds at 60°C, and a final cycle of 15 seconds at 95°C, 1 minute at 60°C and 15 seconds at 95°C.

## Results

**Figure 2.**
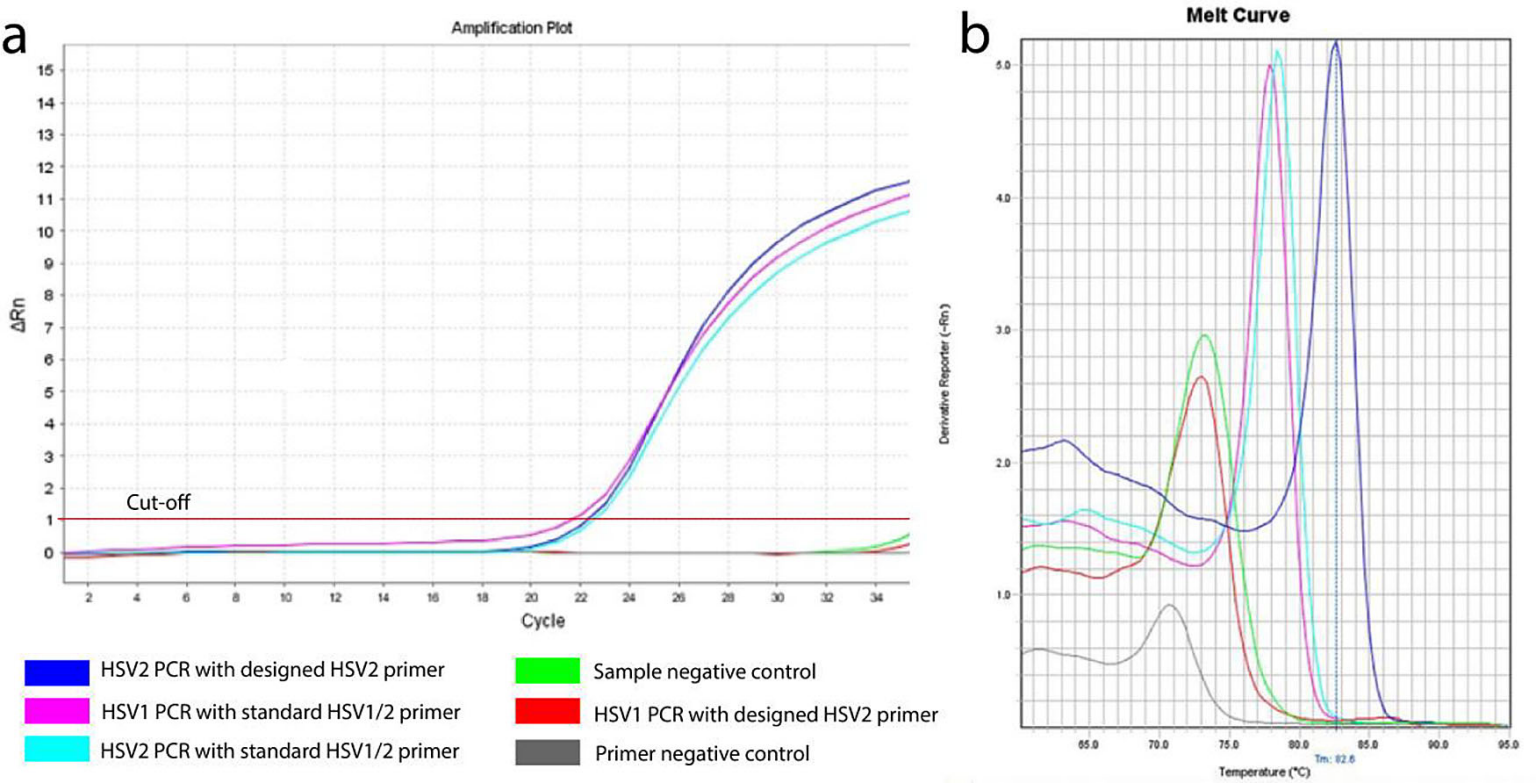
Amplification of HSV2 DNA by RT-PCR. (a) Amplification curves of HSV1 and HSV2 using designed (own) and standard (reference) primers; a lagging stage, logging cycle and plateau are clearly visible; (b) Melt curve of HSV1 and HSV2 using designed (own) and reference (reference) primers.

About 120 base pairs of HSV2 polymerase gene fragment were considered to be amplified using the designed primers, whereas in case of standard control primer pair both HSV1 and HSV2 were amplified ( Figure 2a **)**. The average threshold cycle (Ct) values for reaction by designed and control HSV2 primers were found to be 22.55 and 22.85 respectively, which indicates that the similar initial concentrations of DNA were amplified. ^7, 8^

Observing the melting curve revealed that the designed primer was specific for HSV2. The peak of that amplification was stronger than that of reference primers, indicating the designed primers had a greater sensitivity to the HSV2 target gene than the reference primers ( Figure 2b). ^9, 10^ The melting temperature (Tm) of the amplicon using the designed primers (82.6°C) was also higher than that of the reference primers (about 78°C). The explanation behind this may be a higher GC content of the product or larger fragments than the product amplified by reference primers. ^11^ On the other hand, some unusual peaks for the negative controls were found in the melt curve, which have much higher Ct values (more than 35) and lower Tm of around 70-74°C, which indicates the possibilites of being either non-specific products or primer-dimers. ^12^

In this assay, some secondary non-specific products or primer-dimers were observed in the melt curve, hence, the amplification reaction needs to be optimized. Increasing the annealing temperature of the reaction may reduce non-specific amplification of the product. ^13^ Here, the annealing temperature of the reaction was 60°C; it would be better to use a slightly higher annealing temperature to get only the specific product. However, the annealing temperature should not be too high, as it would reduce the yield of the desired product. ^14^ The optimal annealing temperature can easily be obtained using gradient real-time PCR. ^14^ On the other hand, the concentration of primers can slightly be decreased to prevent the formation of primer-dimer if formed. ^13, 15^

The optimization of Magnesium ion (Mg ^2+^) concentrations is also an important factor to achieve specific and desired product amplification. ^16^ It has been documented that the higher the Mg ^2+^ concentration, the higher the affinity of primers towards hybridization, leading to the formation of primer-dimers and non-specific priming. ^13, 16^ Furthermore, to verify the SYBR Green reaction specificity, an additional agarose gel electrophoresis can be performed using the product and non-specific bands, to confirm the presence of a single PCR product. ^14, 17^ As the SYBR Green fluorescent dye binds with double-stranded DNA and provides fluorescence at the same time as the amplification is running (hence being a real-time assay), all the steps of the protocol should be handled carefully and aseptically. That is why the lack of aseptic conditions may lead to the contamination of the reaction, giving rise to secondary non-specific products. ^18^

## Conclusions

According to the results, the annealing temperature of the reaction should be slightly increased to get specific amplification of HSV2. Furthermore, the primer concentration needs to be decreased to prevent primer-dimer formation. On the other hand, the concentration of Mg ^2+^ in the buffer system can also be reduced to promote the specificity and efficiency of the reaction. Observing the real-time PCR assay, I believe that my designed primers do not need to be altered as the specificity of determining HSV2 is good enough compared with other primers used.

Some modifications to this assay such as proper optimization of the reaction and the use of a probe, specific to that gene, can render it appropriate to be used in a diagnostic laboratory. ^5^ As SYBR Green assays detects all dsDNA, the introduction of a probe (consisting of fluorescent–labeled target-specific oligonucleotides that increase sensitivity and specificity) may establish this assay as a diagnostic test to detect HSV2. Furthermore, to establish this assay as a diagnostic test, positive control with a housekeeping gene (constitutive genes expressed in all cells) is necessary. ^19^ This may promote the efficiency and reliability of this test.

## Data availability

### Underlying data

Figshare: HSV-2 primer and qPCR Ct values, https://doi.org/10.6084/m9.figshare.14603934.v4. ^20^

This project contains the designed HSV2 primer pair and the cycle threshold (Ct) values of HSV2- and HSV1-positive samples generated using qPCR.

NCBI GenBank: Human alphaherpesvirus 2 isolate HSV2_1 DNA polymerase (UL30) gene, complete cds. Accession number: MH697422.1; https://www.ncbi.nlm.nih.gov/protein/MH697422.1.

NCBI GenBank: Human herpesvirus 2 strain RR 60279 DNA polymerase gene, complete cds. Accession number: AY038366.1; https://www.ncbi.nlm.nih.gov/protein/AY038366.1.

NCBI GenBank: HIV-1 clone MNT3-2-12 from USA gag protein (gag) gene, partial cds. Accession number: AY038267.1; https://www.ncbi.nlm.nih.gov/protein/AY038267.1.

NCBI GenBank: Human herpesvirus 2 strain 1605/2007 DNA polymerase (UL30) gene, complete cds. Accession number: JQ352193.1; https://www.ncbi.nlm.nih.gov/protein/JQ352193.1.

NCBI GenBank: Human herpesvirus 2 strain 1541/2008 DNA polymerase (UL30) gene, complete cds. Accession number: JQ352194.1; https://www.ncbi.nlm.nih.gov/protein/JQ352194.1.

NCBI GenBank: Human herpesvirus 2 isolate HSV-2v_pat1 DNA polymerase (UL30) gene, complete cds. Accession number : JX905315.1; https://www.ncbi.nlm.nih.gov/protein/JX905315.1NCBI GenBank: Human herpesvirus 1 UL30 gene for DNA polymerase, complete cds. Accession number: AB072389.1; https://www.ncbi.nlm.nih.gov/protein/AB072389.1.

NCBI GenBank: Human herpesvirus 1 gene for DNA polymerase, complete cds, clone: HSV-1 R98/0. Accession number: AB070847.2; https://www.ncbi.nlm.nih.gov/protein/AB070847.2.

NCBI GenBank: Herpes simplex virus type 1 DNA polymerase gene. Accession number: X04771.1; https://www.ncbi.nlm.nih.gov/protein/X04771.1.

NCBI GenBank: Herpes simplex virus type 1 strain ANG gene for DNA polymerase. Accession number: X04495.1; https://www.ncbi.nlm.nih.gov/protein/X04495.1.

NCBI GenBank: Herpes simplex virus type 1 (KOS) ori-L region and polymerase (pol) gene, complete cds. Accession number: M10792.1; https://www.ncbi.nlm.nih.gov/protein/M10792.1.

NCBI GenBank: Human herpesvirus 1 gene for DNA polymerase, complete cds, clone: HSV-1 R98/3. Accession number: AB070848.2; https://www.ncbi.nlm.nih.gov/protein/AB070848.2.

Data are available under the terms of the Creative Commons Zero “No rights reserved” data waiver (CC0 1.0 Public domain dedication).
