# Supplementary material for: Detection of herpes simplex virus 2: a SYBR-Green-based real-time PCR assay
Source: F1000Res. 2021 Nov 9;10:655. Originally published 2021 Jul 26. [Version 2] doi: 10.12688/f1000research.53541.2 (PMC8634051; doi:10.12688/f1000research.53541.2)
Supplement: Peer review history

# Peer review: Reviewer response for version 2

**DOI:** 10.5256/f1000research.78555.r99494

**Published:** 2021-11-30

**Authors:** Joyanta Modak

**Affiliations:** 1 School of Medicine, Deakin University, Waurn Ponds, VIC, Australia

The authors have addressed all the comments. I don't have any further comments on this manuscript.

Is the rationale for developing the new method (or application) clearly explained?

Partly

Is the description of the method technically sound?

Yes

Are the conclusions about the method and its performance adequately supported by the findings presented in the article?

Yes

If any results are presented, are all the source data underlying the results available to ensure full reproducibility?

Yes

Are sufficient details provided to allow replication of the method development and its use by others?

No

Reviewer Expertise:

My area of expertise is microbiology and molecular biology.

I confirm that I have read this submission and believe that I have an appropriate level of expertise to confirm that it is of an acceptable scientific standard.

# Peer review: Reviewer response for version 2

**DOI:** 10.5256/f1000research.78555.r99493

**Published:** 2021-11-22

**Authors:** ASM Monjur Al Hossain

**Affiliations:** 1 Department of Pharmaceutical Technology, Faculty of Pharmacy, University of Dhaka, Dhaka, Bangladesh

Shaha *et al*. addressed all the issues scientifically. Therefore, the manuscript can be indexed.

Is the rationale for developing the new method (or application) clearly explained?

Yes

Is the description of the method technically sound?

Yes

Are the conclusions about the method and its performance adequately supported by the findings presented in the article?

Yes

If any results are presented, are all the source data underlying the results available to ensure full reproducibility?

Yes

Are sufficient details provided to allow replication of the method development and its use by others?

Yes

Reviewer Expertise:

Drug delivery

I confirm that I have read this submission and believe that I have an appropriate level of expertise to confirm that it is of an acceptable scientific standard.

# Peer review: Reviewer response for version 1

**DOI:** 10.5256/f1000research.56937.r93395

**Published:** 2021-10-05

**Authors:** Joyanta Modak

**Affiliations:** 1 School of Medicine, Deakin University, Waurn Ponds, VIC, Australia

Shaha *et al*., aimed to develop a new primer for the detection of herpes simplex virus 2 using real-time PCR. They used the Primer-BLAST tool to design a primer that can detect only HSV2, not HSV1. However, they are few issues about the manuscript that needs to be addressed.

1. Title of the manuscript – this is not a rapid antigen test using a kit or immunochromatographic test that can be done in 5 minutes. So, the word “Rapid” should remove from the title.
2. There are quite a few published articles that developed a real-time PCR method for the detection of HSV2. In the introduction, authors should acknowledge previously published articles and should describe what is lacking the previous method and why they are developing new methods.
3. What type of samples were used, e.g. clinical sample, extracted DNA, lab strains etc.
4. How many samples were used to validate the method?
5. What are the sensitivity and specificity of the primer?
6. Please provide a table for the new and reference primer.
7. Authors should write results and discussions together instead of results alone.
8. The authors should highlight the limitation of the method such as the number of samples were used.
9. Please delete the word “my” in the conclusion.
10. Authors should consider improving the flow of writing.
11. Please update the references with the most recent articles.

Is the rationale for developing the new method (or application) clearly explained?

Partly

Is the description of the method technically sound?

Yes

Are the conclusions about the method and its performance adequately supported by the findings presented in the article?

Yes

If any results are presented, are all the source data underlying the results available to ensure full reproducibility?

Yes

Are sufficient details provided to allow replication of the method development and its use by others?

No

Reviewer Expertise:

My area of expertise is microbiology and molecular biology.

I confirm that I have read this submission and believe that I have an appropriate level of expertise to confirm that it is of an acceptable scientific standard, however I have significant reservations, as outlined above.

# Response

**Published:** 2021-10-18

**Authors:** Modhusudon Shaha

**Affiliations:** National Institute of Biotechnology, Bangladesh

Thank you so much for your thoughtful and constructive comments on the manuscript.

- *Title of the manuscript – this is not a rapid antigen test using a kit or immunochromatographic test that can be done in 5 minutes. So, the word “Rapid” should remove from the title.*
- **Response: **The title is revised as ‘Detection of herpes simplex virus 2: a SYBR-Green-based real-time PCR assay’.
- *There are quite a few published articles that developed a real-time PCR method for the detection of HSV2. In the introduction, authors should acknowledge previously published articles and should describe what is lacking the previous method and why they are developing new methods.*
- **Response: **The required section is updated as per the reviewer’s recommendation.
- *What type of samples were used, e.g. clinical sample, extracted DNA, lab strains etc.*
- **Response: **We used an HSV2 lab strain and extracted DNA to perform the PCR assay. It has been updated in the Methods section of the manuscript.
- *How many samples were used to validate the method?*
- **Response:** We performed three experiments to confirm the specificity of the used primers to HSV2 strains. We also cross-checked the results with an HSV1 lab strain. The detailed results were described in the Results section of the manuscript.
- *What are the sensitivity and specificity of the primer?*
- **Response:** The primers were of improved sensitivity and specificity compared to the standard primer set that was used to amplify both HSV1 and HSV2 DNAs (described in the Results section). As we tried to declare a new HSV2-specific primer set, we did not determine the sensitivity and specificity of the primers.
- *Please provide a table for the new and reference primer.*
- **Response: **The designed primers used for this study were given in the data availability section.
- *Authors should write results and discussions together instead of results alone.*
- **Response:** The Results section is updated as Results and Discussion.
- *The authors should highlight the limitation of the method such as the number of samples were used.*
- **Response: **The manuscript was updated with the inclusion of the limitation statement.
- *Please delete the word “my” in the conclusion*.
- **Response: **Required section is updated.
  ​​​
- *Authors should consider improving the flow of writing.*
- **Response:** The manuscript was checked again and revised accordingly.

# Peer review: Reviewer response for version 1

**DOI:** 10.5256/f1000research.56937.r91924

**Published:** 2021-09-07

**Authors:** ASM Monjur Al Hossain

**Affiliations:** 1 Department of Pharmaceutical Technology, Faculty of Pharmacy, University of Dhaka, Dhaka, Bangladesh

First, I would like to thank all the authors for their nice write up. The authors explained the methods clearly. In introduction, the authors clearly describe the background of the project. They  sum up the conclusion nicely. They also included necessary citations. However, I have some queries for the researchers:

1. In the methods section, what are the default parameters used in the Primer-BLAST tool for designing the primer? 
2. What is the rationale behind selecting MH697422 as the reference one?
3. What are the sequences of primer-3? 
4. In the results section, have you tried using different Mg++ concentrations, higher melting temp. to check whether the non-specific melting curve was still there or not?

Addressing these points, the manuscript can be indexed.

Is the rationale for developing the new method (or application) clearly explained?

Yes

Is the description of the method technically sound?

Yes

Are the conclusions about the method and its performance adequately supported by the findings presented in the article?

Yes

If any results are presented, are all the source data underlying the results available to ensure full reproducibility?

Yes

Are sufficient details provided to allow replication of the method development and its use by others?

Yes

Reviewer Expertise:

Drug delivery

I confirm that I have read this submission and believe that I have an appropriate level of expertise to confirm that it is of an acceptable scientific standard, however I have significant reservations, as outlined above.

# Response

**Published:** 2021-10-18

**Authors:** Modhusudon Shaha

**Affiliations:** National Institute of Biotechnology, Bangladesh

Thank you so much for your constructive comments on this. To design the appropriate primers, we used the defaults parameters of Primer-BLAST tool of NCBI database. More specifically, max Tm difference of 3 ^o^C (min Tm- 67 ^o^C and max Tm 63 ^o^C), PCR product size of min 70 bp, and max 200 bp. With regards to the use of MH697422 as a reference sequence, this accession contains the complete sequence of DNA polymerase gene of HSV2, on which we were interested.

 The sequences of primer-3 are given in the data availability section which was deposited to Figshare. Additionally, the primer sequences are given below-

 Forward

 GATCTACAAGGTCCCGCTCG

 Reverse

 CACCATCCCGTTCACCTTGA

 Regarding question 4, we used a commercially available PCR master mix (described in the Methods section) for PCR amplification. Therefore, we did not use different Mg++ concentrations.
